# Supplementary material for: Eco-Friendly 1,3-Dipolar Cycloaddition Reactions on Graphene Quantum Dots in Natural Deep Eutectic Solvent
Source: Nanomaterials (Basel). 2020 Dec 18;10(12):2549. doi: 10.3390/nano10122549 (PMC7765906; doi:10.3390/nano10122549)
Supplement: Supplementary file 1 [file nanomaterials-10-02549-s001.pdf]

Electronic Supplementary Material

to

Eco-Friendly 1,3-Dipolar Cycloaddition reactions on Graphene Quantum Dots in Natural Deep Eutectic Solvents

Salvatore V. Giofré,[1] Matteo Tiecco,[2] Consuelo Celesti,[3] Salvatore Patanè,[4] Claudia Triolo,[5] Antonino Gulino,[6] Luca Spitaleri,[6] Silvia Scalese,[7] Mario Scuderi [7] and Daniela Iannazzo[3,*]

[1] *Dipartimento di Scienze Chimiche, Biologiche, Farmaceutiche ed Ambientali, Università di Messina, Viale Annunziata, I-98168 Messina, Italy*

[2] *Dipartimento di Chimica, Biologia and Biotecnologie, Università di Perugia, via Elce di Sotto 8, I-06123 Perugia, Italy.*

[3] *Dipartimento di Ingegneria, Università of Messina, Contrada Di Dio, I-98166 Messina, Italy;*

[4] *Dipartimento di Scienze Matematiche e Informatiche, Scienze Fisiche e Scienze della Terra, Università of Messina, Viale F. Stagno d'Alcontres, 98166 Messina, Italy*

[5] *Dipartimento Ingegneria Civile, Energia e Ambiente, Università "Mediterranea", Loc. Feo di Vito, 89122, Reggio Calabria, Italy*

[6] *Dipartimento di Scienze Chimiche, Università di Catania and I.N.S.T.M. UdR of Catania, Viale Andrea Doria 6, 95125 Catania, Italy*

[7] *Institute for Microelectronics and Microsystems, National Research Council (CNR-IMM), Ottava Strada n.5, I-95121 Catania, Italy.*

** Correspondence to: diannazzo@unime.it; Tel.: +39 090 6766569*

Contents

Figure. S1. Raman spectra (λ_{exc} =532 nm) of pristine MWCNTs and of the synthesized GQDs…S2

Scheme S1. Synthesis of nitrone 2b……………………………………………………..…....S2

Figure S2. Al-Kα excited XPS of the GQDs sample in the C 1sbinding energy region…………..S3

Figure S3. Al-Kα excited XPS of the GQDs sample in the O 1s binding energy region……..…..S3

Figure S4. Size distribution and zeta potential values of *isox*-GQDs 2a and (c) *isox*-GQDs 2b …S4

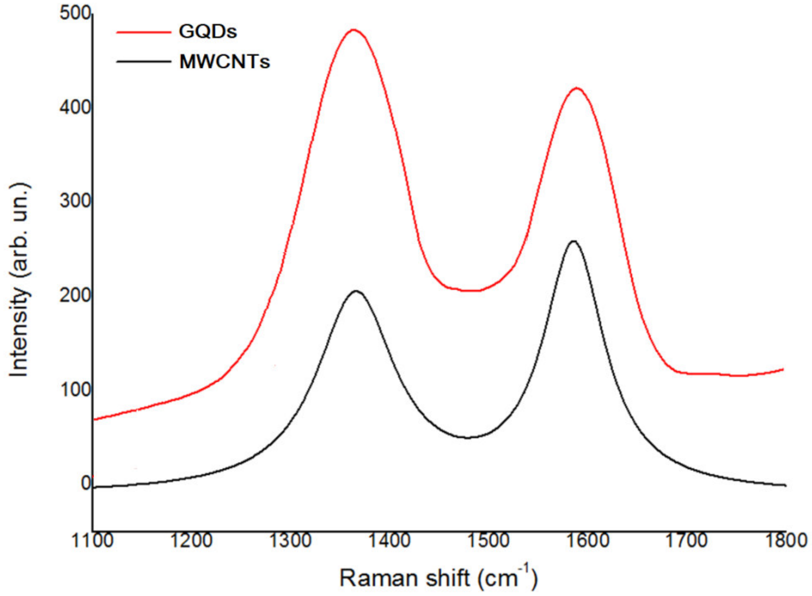

Figure S1. Raman spectra (λ_{exc} =532 nm) of pristine MWCNTs and of the synthesized GQDs.

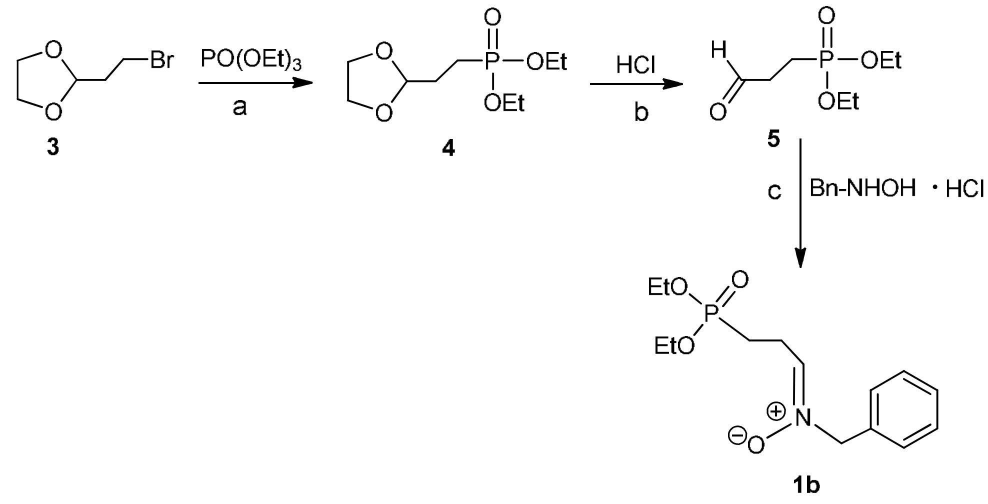

Scheme S1. Synthesis of nitrone 1b. Reagents and conditions: triethyl phosphite (28.9 mmol) 110 °C, 24h (58% yield); (b) 2M HCl 2M, acetone, 3 h at 50 °C (88% yield); (c) sodium acetate, CH_2Cl_2, *N*-benzylhydroxylamine hydrochloride, 1h, 0°C, then 12h, r.t (yield 95%).

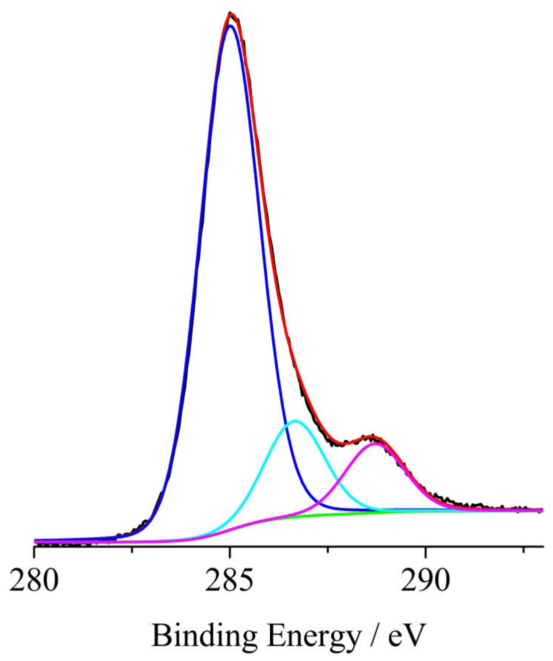

Figure S2. Al-Kα excited XPS of the GQDs sample in the C 1s binding energy region. The blue, cyan and magenta lines refer to the 285.0, 286.7, and 288.7eV Gaussian components; the green line refers to the background and the red line superimposed to the experimental black profile refers to the sum of all Gaussian components.

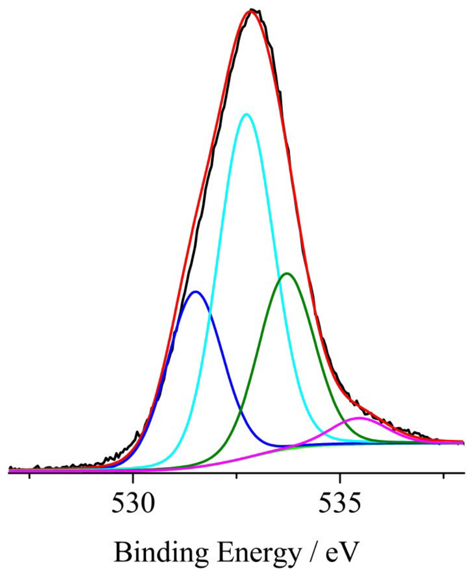

Figure S3. Al-Kα excited XPS of the GQDs sample in the O 1s binding energy region. The black line refers to the experimental profile; the blue, cyan, olive, and magenta lines refer to the 531.5, 532.7, 533.7 and 535.4 eV Gaussian components; the red line, superimposed to the experimental profile, refers to the sum of the Gaussian components.

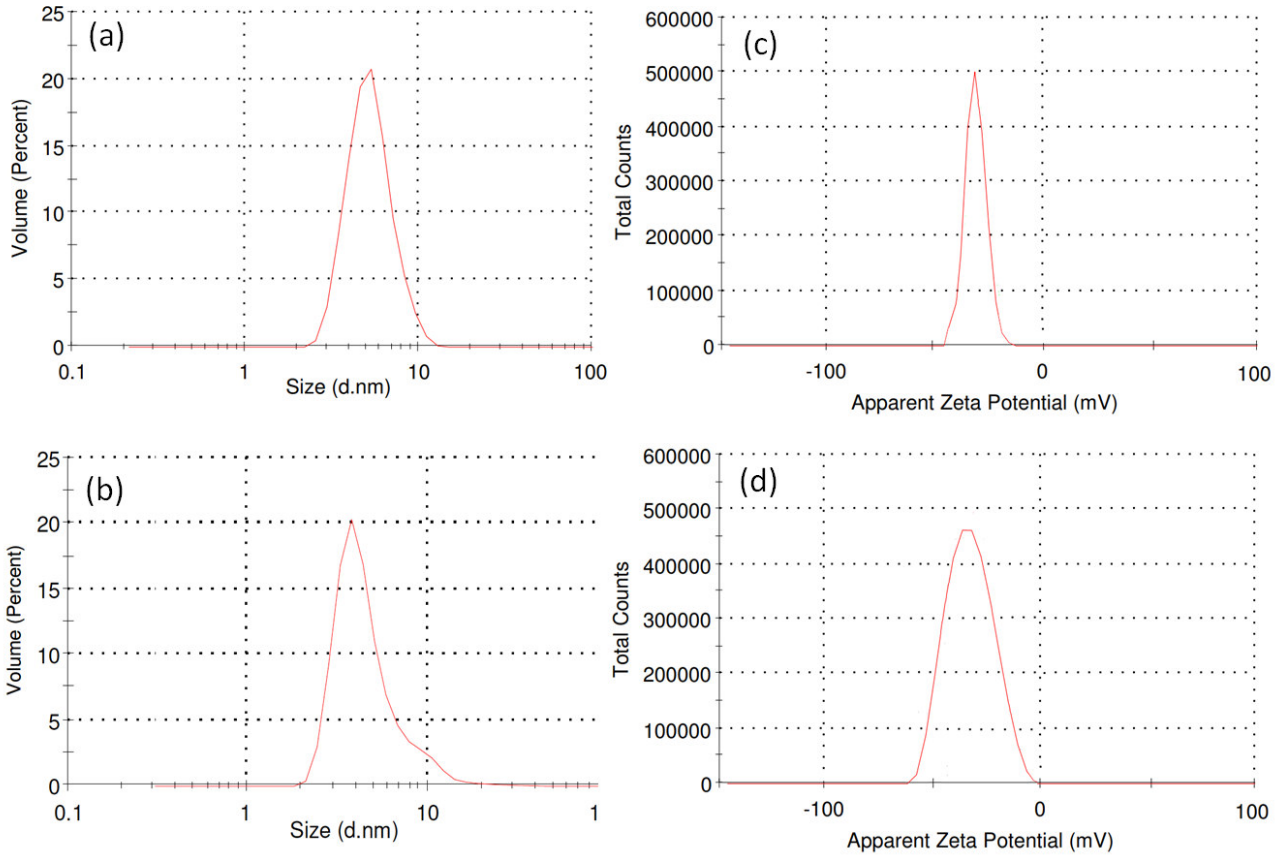

Figure S4. Volume-weighted size distribution of (a) *isox*-GQDs 2a and (c) *isox*-GQDs 2b; zeta potential measurement of (b) *isox*-GQDs 2a and (d) *isox*-GQDs 2b. All the experiments were performed deionized water.
